# Dr. Hercules MacDonnell

**Published:** 1911-06-17

**Authors:** 


### OBITUARY.

The death is announced, in his sixtieth year, of Dr. Hercules MacDonnell, of Dundalk, formerly president of the Irish Medical Association, Surgeon to the County Louth Infirmary and to the County Gaol. The deceased, in addition to his attainments as a surgeon, possessed a calm judgment, much tact, and an intimate knowledge of the Poor Law. He was a fluent speaker.

